# Defecation Warning Monitor Based on ScAlN Piezoelectric Ultrasonic Transducer (PMUT)

**DOI:** 10.3390/mi16050498

**Published:** 2025-04-24

**Authors:** Tao Yao, Jianwei Zong, Haoyue Zhang, Zhiyuan Hou, Liang Lou

**Affiliations:** 1 School of Microelectronics, Shanghai University, Shanghai 201800, China; zhongziqi@shu.edu.cn (T.Y.); zhanghy202408@163.com (H.Z.);; 2 The Shanghai Industrial µTechnology Research Institute, Shanghai 201899, China

**Keywords:** ScAlN, bowel sounds, defecation warning

## Abstract

This study proposes an innovative health management solution to address the defecation care needs of the elderly population. Traditional post-defecation care methods have significant limitations, particularly imposing a considerable psychological burden on patients. By leveraging the rich physiological information contained in bowel sounds, in this work, we designed and implemented a wearable defecation warning monitor based on scandium aluminum nitride (ScAlN) piezoelectric thin films and piezoelectric micromachined ultrasonic transducers (PMUTs). The proposed device mainly incorporates two core components: a bowel sound signal acquisition module and a real-time signal display graphical user interface (GUI) developed using the MATLAB R2023a platform. The research focuses on the systematic characterization and comparative analysis of the sound pressure sensitivity of three different signal readout structures. Experimental results demonstrate that the differential readout structure exhibits superior sensitivity. By continuously monitoring bowel sounds in healthy subjects both with and without the urge to defecate using the defecation warning monitor and a modified stethoscope, and conducting a comparative analysis of the experimental data, it is verified that the defecation warning monitor has significant advantages in clinical applications and demonstrates promising potential for defecation warning monitoring.

## 1. Introduction

The detrimental effects of population aging on social structures have become increasingly evident. With progressive declines in physiological functions, an increasing proportion of older patients experience difficulties in bowel care. According to a clinical study data, there is a significant difference in the incidence of fecal incontinence among elderly patients who have to be hospitalized due to medical needs, ranging from 17% to 66% [1]. Notably, advanced age (particularly over 80 years), neurological disorders, prolonged bedridden status, and polypharmacy correlate with higher incidence rates, often approaching the upper limit of this range. This phenomenon highlights the importance of defecation care in clinical nursing of hospitalized elderly patients.

Defecation care comprises pre-defecation and post-defecation components. Hospitals more commonly implement post-defecation care, which involves fecal collection devices, absorbent products, or specialized beds. However, these reactive measures cause significant psychological distress, and prolonged skin exposure to feces increases infection risks [2]. In contrast, pre-defecation care may prevent skin infections, allow better preparation for healthcare providers, and maintain patient dignity. Developing effective pre-defecation care devices requires accurate monitoring of defecation intentions.

The advancement of Micro-Electro-Mechanical Systems (MEMS) technology has significantly propelled the field of ultrasonic transducers. Micro-machined ultrasonic transducers (MUTs), owing to their advantages in production cost and performance, have been widely applied in numerous domains. As the core of MEMS-based acoustic sensing technology, MUTs primarily include capacitive micro-machined ultrasonic transducers (CMUTs) and piezoelectric micro-machined ultrasonic transducers (PMUTs). The former relies on parallel-plate capacitors for electroacoustic energy conversion, while the latter utilizes the direct and inverse piezoelectric effects of piezoelectric materials for energy transduction. CMUT is significantly affected by parasitic capacitance [3] and requires high bias voltage. Despite their advantages such as high detection sensitivity and wide operating bandwidth, they still face many limitations in miniaturization and precise detection. In contrast, PMUT is not limited by the condition of high bias voltage, with a compact structure and low power consumption, making it increasingly prominent in modern ultrasound sensing applications. PMUTs have a wide range of applications, including gas flow measurement [4,5], solid defect detection [6], MEMS hydrophones [7], ultrasonic echo ranging [8], and wireless energy transfer [9]. In related research, Kim et al. designed a wearable intestinal alert system based on bowel sound acquisition using polyvinylidene fluoride (PVDF) piezoelectric thin-film sensors [10]. However, compared to aluminum nitride (AlN), PVDF is more susceptible to moisture and UV exposure, leading to the gradual degradation of its piezoelectric properties over extended use. As an inorganic material, AlN demonstrates a significantly longer operational lifetime. Additionally, PVDF’s piezoelectric coefficients are sensitive to temperature and other environmental factors. In contrast, although AlN has a smaller piezoelectric coefficient, it demonstrates better stability within a certain temperature range. Nakanishi and colleagues developed a bowel movement detection device that utilizes an ultrasonic sensor to monitor rectal conditions [11]. The system works by receiving and comparing ultrasonic echo signals to detect changes in rectal thickness. However, ultrasound-based intestinal monitoring cannot provide real-time sensing, which limits its practicality for daily monitoring.

This research introduces a novel wearable defecation warning monitor utilizing PMUT technology. The device demonstrates exceptional sensitivity (−151 ± 1 dB) and enables the continuous real-time monitoring of intestinal sounds, allowing the timely detection of defecation urges. The system architecture comprises two main components: (1) a sensor module integrating signal conditioning circuits and a PMUT array, and (2) a MATLAB-based graphical user interface for data visualization and storage via a DAQ (Data Acquisition) card. Validation experiments were conducted on healthy male subjects under two physiological conditions: with and without defecation urges. Both the proposed PMUT-based defecation warning monitor and a modified electronic stethoscope were employed for comparative assessment. The experimental results demonstrate the technical feasibility of the proposed system, showing significant potential for daily bowel activity monitoring.

## 2. Working Principle

### 2.1. A Mathematical Model of Intestinal Sounds

**Figure 1:**
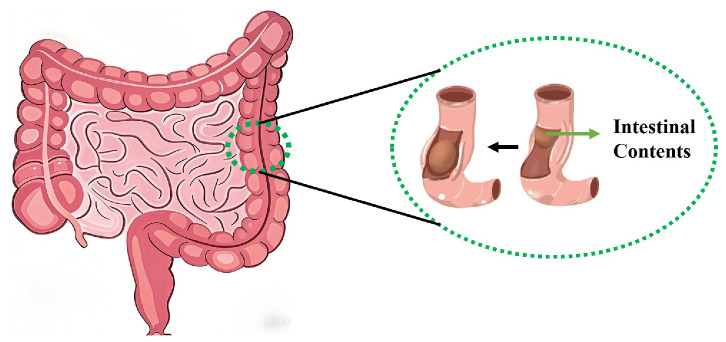
Intestinal peristalsis.

The human bowel sounds primarily originate from intestinal peristalsis, which refers to the symmetrical contraction and relaxation of intestinal muscles that propel intestinal contents, generating characteristic acoustic signals during this process. These sounds propagate through the gastrointestinal system in a wave-like pattern [12]. Based on the bowel sound model proposed by Du [13], intestinal peristalsis is caused by the contraction of intestinal muscles, which compress the intestinal contents into a small segment of the intestine, as illustrated in Figure 1. The fundamental component of bowel sounds is the individual wave, representing the compression of intestinal contents under luminal contraction forces, which reflects the dynamic characteristics of intestinal motility. The physiological mechanism involves cyclic muscular activity: intestinal smooth muscles generate contractile forces that narrow the lumen and compress the contents, followed by muscle relaxation allowing lumen recovery, before initiating a new contraction phase to complete the motility cycle. Each movement cycle consists of two distinct phases—contraction and relaxation—that collectively enable the directional propulsion of contents through the digestive tract. The resonant frequency of bowel sounds can be calculated using Equation (1):

(1) $$M\frac{d^{2}x}{dt^{2}}+C\frac{dx}{dt}+Kx=p$$

where *x* represents the wall motion, *p* denotes the fluid pressure, *M* and *K* are the wall mass and stiffness, respectively, and *C* is the damping coefficient related to energy dissipation. Therefore, the resonant frequency of individual wave ($f_{\mathrm{iwc}}$) components can be given by Equation (2):

(2) $$f_{\mathrm{iwc}}=\frac{1}{2\pi }\sqrt{\frac{k}{M}-\frac{C}{2m^{2}}}$$

Thus, as bowel sound signals exhibit distinct variations corresponding to different intestinal physiological states, their acoustic features can be used to stage-specifically characterize intestinal conditions.

### 2.2. Defecation Warning Monitor

**Figure 2:**
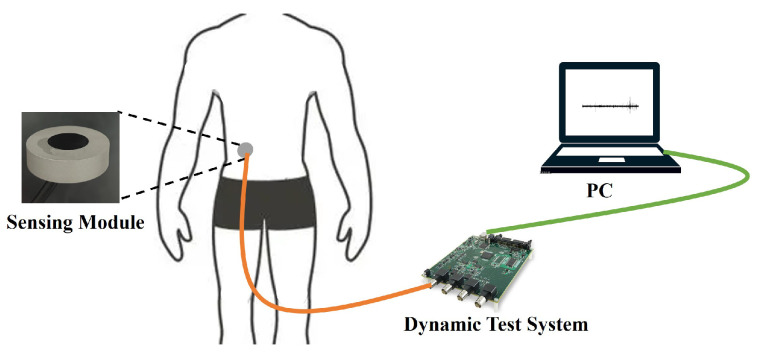
Defecation warning monitor.

The monitoring system in this study is structured as shown in Figure 2, consisting of both hardware and software components. The hardware section includes a sensing module, a data acquisition card (MCC USB-2020), and a computer terminal (PC), which work together to achieve the acquisition and transmission of bowel sound signals. The software system utilizes a MATLAB-based graphical user interface (GUI) and processes the bowel sound signals in real time using a second-order Butterworth bandpass filter (passband: 100–1200 Hz) for visualization. During experiments, the sensing module is first securely attached to a designated position on the subject’s abdomen. The collected bowel sound signals are transmitted to the computer via the data acquisition card, after which the system displays the time-domain waveform in real time.

## 3. Design and Fabrication of the Sensing Module

### 3.1. Principle and Design of PMUT

The receiving performance of PMUTs plays a crucial role in sensing modules. Table 1 summarizes the key material properties of commonly used piezoelectric thin films for PMUT applications. Although PZT exhibits the highest $e_{31,f}$ coefficient, its incompatibility with standard complementary CMOS fabrication processes limits its application. In addition, the biomedical application of PZT (lead zirconate titanate) requires careful consideration of its potential toxicity due to its lead content. In comparison to AlN, ScAlN demonstrates a significantly larger $e_{31,f}$. Moreover, PMUTs fabricated with ScAlN show higher electromechanical coupling coefficients than those made with AlN under identical conditions [14]. This enhanced performance leads to superior receiving sensitivity in ScAlN-based PMUTs compared to their AlN-based counterparts. Therefore, ${\mathrm{Sc}}_{0.2}{\mathrm{Al}}_{0.8}N$ was selected as the piezoelectric transduction layer for the PMUTs in this study.

When operating in receiving mode, incident acoustic waves induce mechanical vibrations in the PMUT’s membrane structure. These vibrations simultaneously generate both tensile and compressive stresses within the piezoelectric functional layer [18]. The curvature distribution of the fixed support beams or diaphragm structure exhibits a characteristic stress inversion node. This node demarcates alternating regions of tension (positive charge accumulation) and compression (negative charge accumulation) on the upper surface of the piezoelectric material, resulting in spatially opposite charge distributions. In this study, we implement a differential electrode configuration by dividing the PMUT’s top electrode into an inner electrode (IE) and an outer electrode (OE). The traditional single-electrode configuration of PMUTs limits charge accumulation to a single region, where *Q* represents the total generated charge and $C_{0}$ denotes the capacitance. In this case, the accumulated charge *Q* on the top electrode yields an output voltage $V_{\mathrm{single}}$ that can be expressed by Equation (3):

(3) $$V_{\mathrm{single}}=\frac{Q}{C_{0}}$$

In the differential electrode configuration, the PMUT simultaneously utilizes both charge accumulation regions with −Q and *Q* charges accumulating at the IE and OE, respectively. The output voltage $V_{\mathrm{diff}}$ in the differential electrode configuration can be expressed by Equation (4):

(4) $$V_{\mathrm{diff}}=\frac{Q}{C_{0}}-\frac{-Q}{C_{0}}=2V_{\mathrm{single}}$$

Therefore, compared to the single-electrode configurations, the differential electrode configuration exhibits a significantly higher output voltage in receiving mode, thereby further optimizing the PMUT’s receiving performance. Poor geometric design of the top electrode can significantly reduce charge collection efficiency.

**Figure 3:**
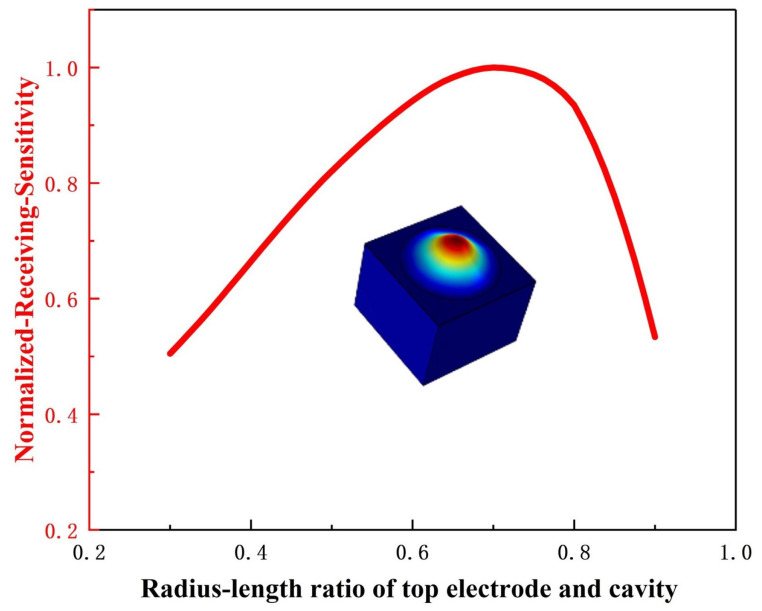
The influence of the radius–length ratio of the top electrode and cavity on the sensitivity of PMUT.

**Figure 4:**
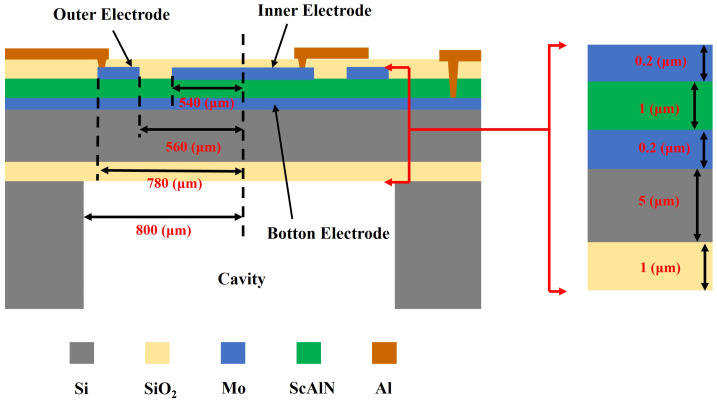
Schematic cross-sectional view of the PMUT.

To optimize the performance of the PMUT, this study employs the finite element simulation software COMSOL Multiphysics 6.1 for modeling. In the simulation, the cavity radius was fixed, and only a single top electrode was used while keeping all other parameters unchanged [19]. A parametric sweep was performed on the radius of the top electrode, and the results are shown in Figure 3. Through comparative analysis, the optimal ratio between the top electrode radius and the cavity radius was determined. When designing a PMUT with a differential electrode configuration, the rationality of electrode distribution was taken into consideration. Accordingly, the IE radius was set to 540 µm, and the outer electrode width was set to 220 µm with a 20 µm gap between them. The geometric structure and parameters of the PMUT are illustrated in Figure 4.

### 3.2. PMUT Manufacturing Industrial Process

**Figure 5:**
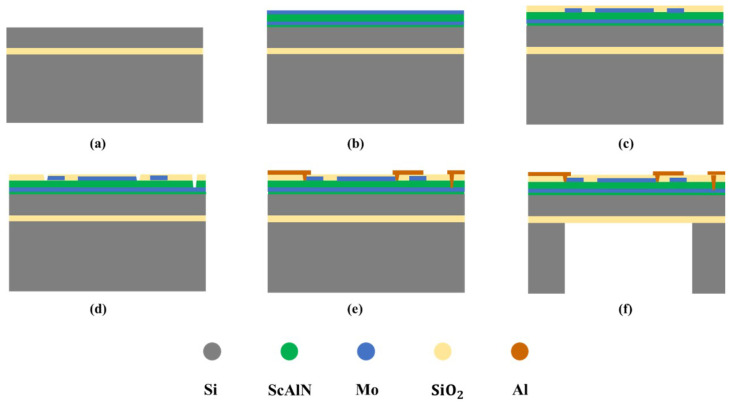
Fabrication process flow of PMUT. (**a**) Single-side polished SOI wafer. (**b**) Deposit Mo bottom electrode layer, AlN piezoelectric layer, and Mo top electrode layer. (**c**) Pattern the top electrode and deposit an oxide insulating layer (SiO_2_). (**d**) Etch electrode vias to connect the top and bottom electrodes. (**e**) Deposit Al electrodes and pattern them to form bonding pads for wire bonding. (**f**) the diaphragm is released by performing backside deep reactive ion etching (DRIE) of the SOI wafer to form the cavity structure.

The complete MEMS manufacturing process used in this study is shown in Figure 5. The process starts with the preparation of a single-side polished SOI wafer (Figure 5a). As illustrated in Figure 5b, a 50 nm ScAlN seed layer with 9.6% doping was deposited via Physical Vapor Deposition (PVD), which was followed by the deposition of a 0.2 µm Mo bottom electrode layer, a 1 µm ScAlN piezoelectric layer, and a 0.2 µm Mo top electrode layer. As shown in Figure 5c, the top electrode was etched using reactive ion etching (RIE), and after patterning the top electrode, an oxide insulating layer (${\mathrm{SiO}}_{2}$) was deposited via Plasma-Enhanced Chemical Vapor Deposition (PECVD). As depicted in Figure 5d, electrode vias were etched using RIE to facilitate connections between the top and bottom electrodes. As illustrated in Figure 5e, Al aluminum electrodes were deposited via PVD and patterned to form electrical interconnects. In the final step (Figure 5f), the diaphragm is released by performing backside deep reactive ion etching (DRIE) of the SOI wafer to form the cavity structure.

**Figure 6:**
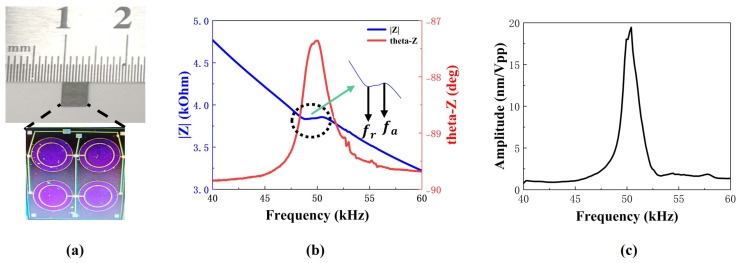
(**a**) Optical images of PMUT array. (**b**) Electrical properties of PMUT array. (**c**) Measurement of the PMUT array’s frequency response using LDV.

The prepared PMUT array was observed using an optical microscope, and the results are shown in Figure 6a. The PMUT array is arranged in a 2 × 2 distribution with the final chip size measuring 3.8 mm × 4.4 mm.

The effective electromechanical coupling coefficient ($k_{\mathrm{eff}}^{2}$) is a critical performance parameter of PMUTs that quantifies the energy conversion efficiency between mechanical and electrical domains. When operating in receiver mode, this parameter becomes particularly crucial, as enhanced $k_{\mathrm{eff}}^{2}$ values directly improve electromechanical transduction efficiency, consequently enhancing receiving sensitivity. The calculation of $k_{\mathrm{eff}}^{2}$ is given in Equation (5) [20]:

(5) $$k_{\mathrm{eff}}^{2}=1-{\left(\frac{f_{r}}{f_{a}}\right)}^{2}$$

where $f_{a}$ is the anti-resonance frequency, and $f_{r}$ is the resonance frequency of the piezoelectric material. Impedance measurements of the fabricated PMUT array were conducted using an impedance analyzer (E4990A, KeysightTechnologies, SantaRosa, CA, USA). The corresponding results are displayed in Figure 6b. The $f_{r}$ measurement of the PMUT array is 48.88 kHz, and the $f_{a}$ is 50.45 kHz. According to Equation (5), the $k_{\mathrm{eff}}^{2}$ is calculated to be 6.1%.

The mechanical vibration performance of the PMUT device was characterized using a laser Doppler vibrometer (MSA 600, Polytec GmbH, Waldbronn, Germany). The test results, as shown in Figure 6c, were obtained by applying a sinusoidal excitation signal with a frequency sweep (40 kHz to 60 kHz, 1 Vpp). The first-order resonance frequency of the PMUT was found to be approximately 50 kHz with a peak displacement of 19.45 nm/Vpp.

### 3.3. Encapsulation and Testing of the Sensing Module

**Figure 7:**
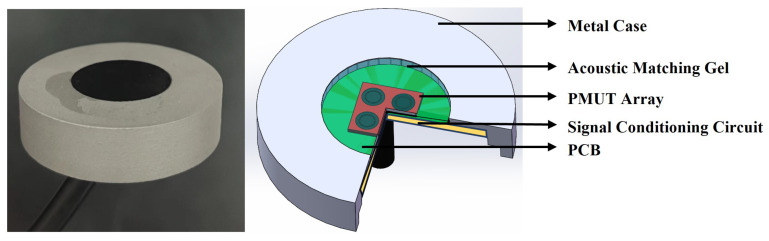
Schematic diagram of sensing module and packaging of sensing module.

The sensing module comprises a PMUT array, a preamplifier, an acoustic matching gel, a 316L stainless steel housing, and an electronic packaging adhesive. The encapsulated sensing module is shown in Figure 7; the top layer consists of an acoustic matching gel (polyurethane) for impedance coupling, followed by the PMUT array and signal conditioning circuit in the middle, while the bottom layer uses a silicone potting compound for electronic encapsulation and mechanical fixation. The selection of acoustic matching gel plays a decisive role in the encapsulation design of the sensing module, as its performance directly impacts both the acquisition quality of bowel sounds and system reliability. When detecting bowel sounds, the packaging material must simultaneously meet dual requirements of acoustic performance and physical protection: it must achieve precise acoustic impedance matching (typically within 1.5–1.7 MRayl) to ensure efficient signal transmission from biological tissues to the sensor while minimizing signal reflection and energy loss caused by acoustic impedance mismatch at media interfaces, while also providing excellent sealing properties to prevent bodily fluid infiltration and external interference, along with good biocompatibility for long-term wearable or implantable applications. The acoustic transmission coefficient (*T*) at the material interface can be calculated using Equation (6) [21]:

(6) $$T=\frac{4Z_{1}Z_{2}}{{\left(Z_{1}+Z_{2}\right)}^{2}}$$

where $Z_{1}$ and $Z_{2}$ represent the characteristic acoustic impedances of the adjacent media at the interface. The average acoustic impedance of human soft tissue is approximately 1.63 MRayl. According to Equation (6), the impedance coefficients of the two materials should be as close as possible to improve transmission efficiency. An acoustic matching material with an impedance of 1.57 MRayl was selected. The packaged sensor module is shown in the figure with dimensional specifications of 38 mm × 10 mm.

**Figure 8:**
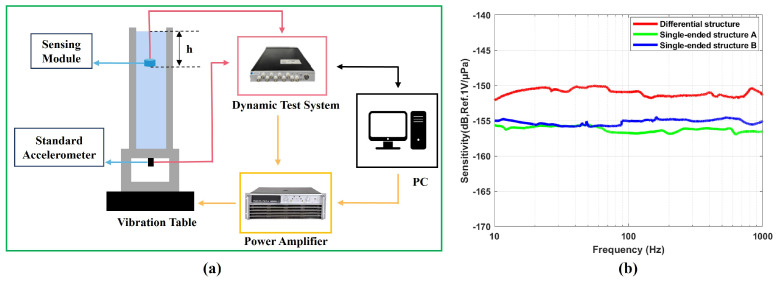
(**a**) Vibrating liquid column measurement system. (**b**) Sensitivity curves of three different readout structures.

This study implemented three distinct signal readout configurations based on the signal conditioning circuit: differential structure, single-ended structure A, and single-ended structure B. In the differential structure, the signal conditioning circuit is connected to both the IE and OE, while the bottom electrode is grounded. In single-ended structure A, the bottom electrode and OE are connected to the signal conditioning circuit with the IE left floating. In single-ended structure B, the bottom electrode and IE are connected to the signal conditioning circuit, while the OE remains floating. This work evaluated the sensing module’s sensitivity using the experimental configuration illustrated in Figure 8a. The sensing module under test was first fixed at a predetermined height h above the liquid surface. Subsequently, the acceleration and frequency of the vibration signal were set, and the vibration signal was amplified by a power amplifier before being output to the shaker. At the last stage, the sensing module transmits the electrical signal to the dynamic test system. The sensitivity ($S_{M}$) of the sensing module was calculated using Equation (7) [22]:

(7) $$S_{M}=\frac{U_{M}}{U_{A}}\times \frac{S_{A}}{\rho_{h}}$$

Here, $U_{m}$ and $U_{A}$, respectively, represent the output voltage of the sensing module and the parameter, $S_{A}$ denotes the standard accelerometer sensitivity, and ρ is the density of the liquid. Three different sensing modules with different readout structures were tested, as shown in Figure 8b. They all exhibited very flat responses within the operating frequency range (10 Hz to 1000 Hz). The receiving sensitivity of the differential structure was −151 ± 1 dB, while the receiving sensitivity of the single-ended structure A and single-ended structure B were −156 ± 1 dB and −155 ± 1 dB, respectively. The experimental results showed that compared to the single-ended structure, the differential structure had higher sensitivity. Table 2 provides a performance comparison between the manufactured sensing module and existing related research.

The noise resolution becomes a critical parameter when detecting weak bowel sounds using the sensing module, as both the desired signals and noise are amplified by the signal conditioning circuit. This parameter can be calculated using Equation (8) [26]:

(8) $$\mathrm{NoiseResolution}=\frac{\mathrm{Noisefloor}}{\mathrm{Sensitivity}}$$

**Figure 9:**
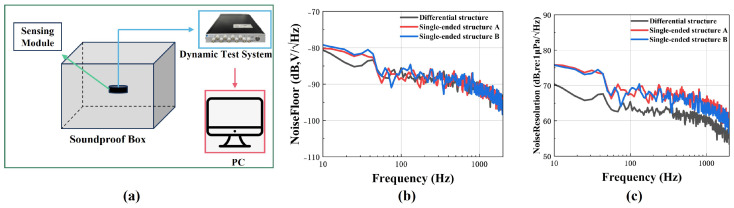
(**a**) Noise measurement system. (**b**) Noise density of three different readout structures. (**c**) Noise resolution of three different readout structures.

Figure 9a shows the experimental setup used to measure the baseline noise levels for all readout configurations. The measurements were conducted in an anechoic chamber (Figure 9b), and all three readout configurations exhibited comparable noise floor levels. According to Equation (8) and Figure 9c, the differential PMUT structure achieved a noise resolution of 62.89 dB at 500 Hz, while the single-ended PMUT structures A and B demonstrated noise resolutions of 68.45 dB and 67.18 dB, respectively, at the same frequency. Consequently, the differential PMUT structure exhibits lower noise gain compared to the single-ended PMUT structures. Taking into account both sensitivity and noise resolution, this study ultimately selected the differential structure as the signal readout configuration for the PMUT.

## 4. Experiments and Discussion

**Figure 10:**
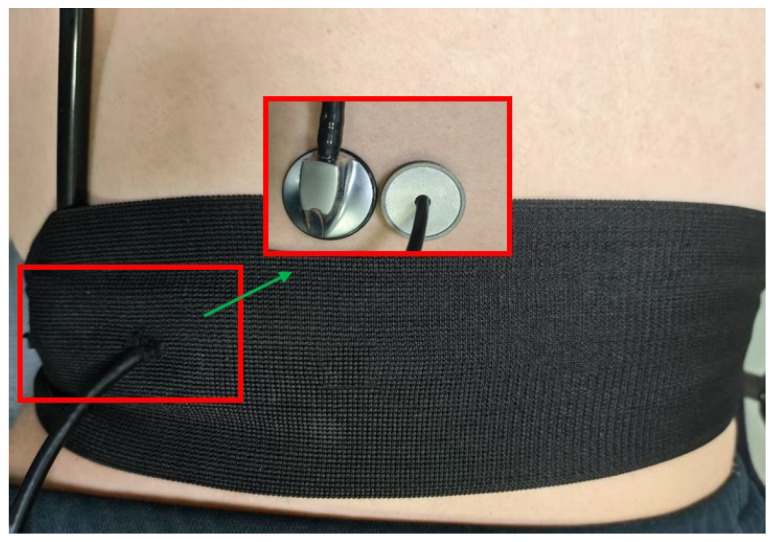
Experimental setup for defecation monitoring.

In order to test the performance of the defecation warning monitor, we compared a modified stethoscope with the defecation warning monitor. The modified stethoscope was developed by integrating an electret condenser microphone module into the traditional stethoscope structure with the microphone module embedded in the rubber sound tube of the stethoscope. Three healthy adult male volunteers (A, B, and C) participated in this study. The experiment is divided into two stages: the first stage is to monitor the subjects continuously for 4 min when they have the urge to defecate and then rest for 60 min after defecation. The second stage includes continuous monitoring for 16 min under non-defecation impulse conditions. The collected data are processed over a 4-min period. Before the experiment, the designed sensing module and modified stethoscope were tightly attached to the abdominal wall of the human body and fixed, as shown in Figure 10. Throughout the experiment, subjects maintained a supine position while minimizing the generation of noise.

**Figure 11:**
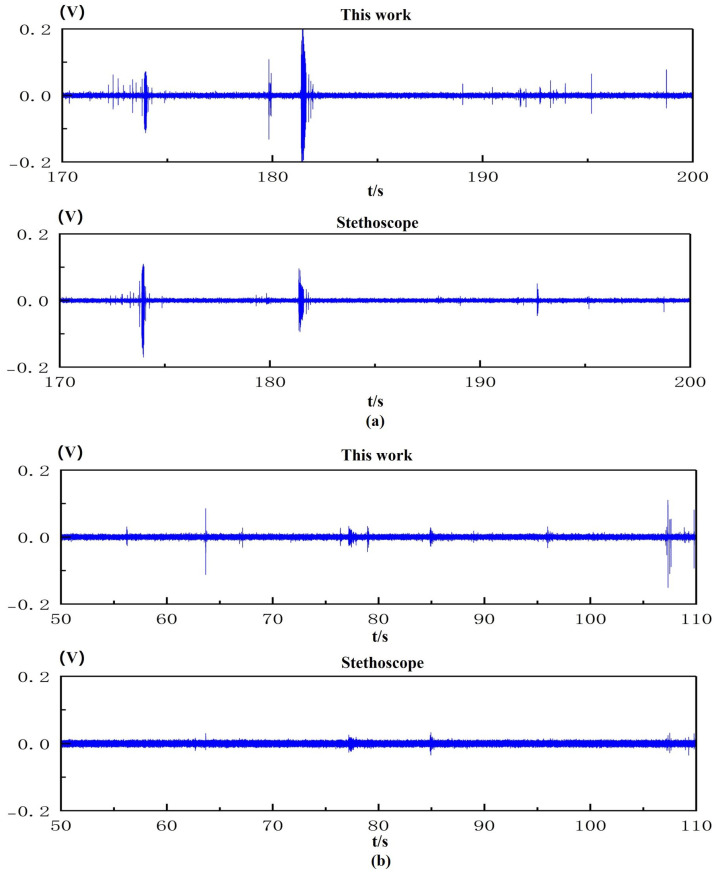
(**a**) Bowel sounds measured using the defecation warning monitor and modified stethoscope when the urge to defecate is present. (**b**) Bowel sounds measured using the defecation warning monitor and modified stethoscope when the urge to defecate is absent.

**Figure 12:**
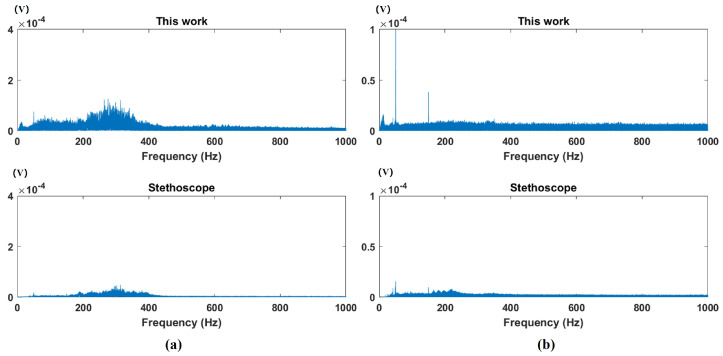
(**a**) Spectral analysis of bowel sounds when the urge to defecate is present. (**b**) Spectral analysis of bowel sounds when the urge to defecate is absent.

Figure 11a,b present the bowel sound monitoring results obtained by the proposed monitoring device and the modified stethoscope under defecation-urge and non-urge conditions, respectively. This study employed a controlled experimental design to systematically compare the performance differences in bowel sound acquisition between the PMUT-based defecation warning monitor and the modified stethoscope. The experimental data clearly demonstrate that the PMUT-based defecation warning monitor exhibits significant superiority in time-domain signal acquisition capabilities. As shown in Figure 11, its outstanding high-sensitivity characteristics enable the precise detection and recording of more low-intensity bowel sound signals. Spectral analysis results (Figure 12a,b) reveal that the bowel sound signals exhibit distinctive frequency-domain characteristics with their core frequency components predominantly concentrated in the specific range of 100–500 Hz. This spectral feature shows remarkable consistency with previously reported bowel sound frequency characteristics by Ranta et al. [27], further validating the stability of acoustical signals generated by intestinal motility in the frequency domain. These findings not only confirm the reliability of previous research but, more importantly, provide critical parameter references for the frequency-band optimization design of bowel sound monitoring devices.

**Figure 13:**
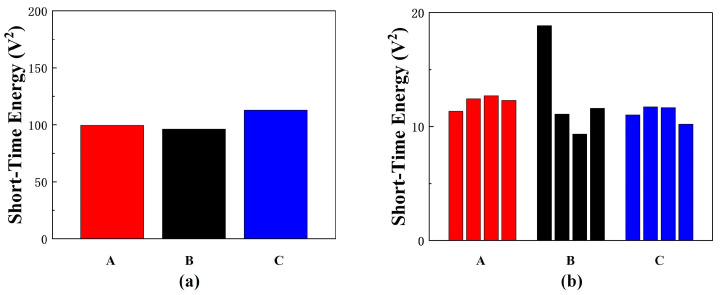
(**a**) Short-term energy of bowel sounds recorded by the defecation warning monitor from three volunteers during defecation urge states, presented in 4-min segments. (**b**) Short-term energy of bowel sounds recorded by the defecation warning monitor from three volunteers under non-defecation urge conditions, presented in 4-min segments.

Through short-time energy analysis, as shown in Figure 13, it was found that intestinal activity exhibits higher levels of activity when the urge to defecate is present, while activity is relatively lower when the urge is absent. This may be due to the more pronounced intestinal peristalsis triggered as the colon pushes feces into the rectum.

## 5. Conclusions and Future Work

This study introduces a highly sensitive, wearable defecation warning monitor utilizing a PMUT, which can be used for bowel movement monitoring in elderly patients. The piezoelectric material employed is ScAlN with an effective electromechanical coupling coefficient of 6.1%. Three different readout structures were experimentally tested, and the results demonstrated that the differential readout structure offers higher sensitivity compared to the single-ended readout structures. The reported defecation warning monitor consists of two modules: one for acquiring bowel sounds and another for displaying the bowel sounds in real time through a graphical user interface. Experiments were conducted on healthy adult male subjects under two conditions: with and without the urge to defecate. During the monitoring experiments, compared to the modified stethoscope, the developed sensing module was able to capture relatively weak bowel sound signals, which are crucial for bowel movement monitoring, as they may help elucidate the relationship between defecation and bowel sounds. The experimental results indicate that the developed sensing module achieves effective information acquisition in bowel movement monitoring. In future studies, we will expand the participant cohort to ensure demographic diversity and develop an automated bowel movement alert algorithm to achieve intelligent defecation monitoring.

## Figures and Tables

**Table 1:** Properties of commonly used piezoelectric materials.

Properties	PZT [15]	AlN [16]	${\mathrm{Sc}}_{0.2}{\mathrm{Al}}_{0.8}N$ [17]
$e_{31,f}\;\left({C/m}^{2}\right)$	−10	−1.05	−1.51
$\varepsilon_{33}\;\left(F/m\right)$	1200	10.5	12.31
$e_{31,f}/\varepsilon_{33}$	−0.008	−0.1	−0.123

**Table 2:** Comparison of the stethoscope devices in this study with existing research.

Author	Chip Size (mm)	Sensitivity (dB)
Chongbin Liu [23]	3.2 × 3.2	−154 ± 0.5
Licheng Jia [24]	3.2 × 3.2	−178
Yuhua Yang [25]	N/A	−180.6
This work	3.8 × 4.4	−151 ± 1

## Data Availability

Data are contained within the article.
